# Laser-Induced Graphene Stretchable Strain Sensor with Vertical and Parallel Patterns

**DOI:** 10.3390/mi13081220

**Published:** 2022-07-29

**Authors:** Yu-Hsin Yen, Chao-Shin Hsu, Zheng-Yan Lei, Hsin-Jou Wang, Ching-Yuan Su, Ching-Liang Dai, Yao-Chuan Tsai

**Affiliations:** 1 Department of Bio-Industrial Mechatronics Engineering, National Chung Hsing University, Taichung City 402, Taiwan; cefb2221@gmail.com (Y.-H.Y.); robin051311@gmail.com (C.-S.H.); jm21611294@gmail.com (Z.-Y.L.); a29555623@gmail.com (H.-J.W.); 2 Smart Sustainable New Agriculture Research Center (SMARTer), Taichung City 402, Taiwan; cldai@dragon.nchu.edu.tw; 3 Graduate Institute of Energy Engineering, National Central University, Taoyuan City 320, Taiwan; cysu@ncu.edu.tw; 4 Department of Mechanical Engineering, National Chung Hsing University, Taichung City 402, Taiwan

**Keywords:** laser-induced graphene, stretchable strain sensor, gauge factor, polymer carbonization

## Abstract

In intelligent manufacturing and robotic technology, various sensors must be integrated with equipment. In addition to traditional sensors, stretchable sensors are particularly attractive for applications in robotics and wearable devices. In this study, a piezoresistive stretchable strain sensor based on laser-induced graphene (LIG) was proposed and developed. A three-dimensional, porous LIG structure fabricated from polyimide (PI) film using laser scanning was used as the sensing layer of the strain sensor. Two LIG pattern structures (parallel and vertical) were fabricated and integrated within the LIG strain sensors. Scanning electron microscopy, an X-ray energy dispersive spectrometer, and Raman scattering spectroscopy were used to examine the microstructure of the LIG sensing layer. The performance and strain sensing properties of the parallel and vertical stretchable LIG strain sensors were investigated in tensile tests. The relative resistance changes and the gauge factors of the parallel and vertical LIG strain sensors were quantified. The parallel strain sensor achieved a high gauge factor of 15.79 in the applied strain range of 10% to 20%. It also had high sensitivity, excellent repeatability, good durability, and fast response times during the tensile experiments. The developed LIG strain sensor can be used for the real-time monitoring of human motions such like finger bending, wrist bending, and throat swallowing.

## 1. Introduction

In recent years, the demand for portable and wearable devices with sensing functions has rapidly increased. The growing demand for these devices is expected to lead to the development of promising technologies for fabricating stable sensors. Various sensors for converting external stimuli into readable electrical signals have been proposed and used widely in robots and wearable medical devices. Strain sensors are a key type of sensor used to monitor complex human actions, such as finger or wrist bending, in many medical applications [1,2,3,4]. Strain sensors can provide real-time health information on heartbeat, respiration, pulse, or other biological functions to monitor physical exertion or for medical diagnosis. Strain sensors have excellent potential for use in healthcare devices that pick up minute physiological signals [5,6,7,8,9,10].

Conventional strain sensors usually comprise silicon substrate or rigid materials. However, sensors produced with these materials have a small strain measurement range; this limited deformation reduces the applicability of strain sensors in various fields. To increase the strain measurement range, sensors with stretchable substrates have been proposed. Many such sensors comprise an elastic substrate, a sensing layer, and a pair of electrodes [11]. Polydimethylsiloxane (PDMS) is widely employed as an elastic substrate due to its excellent mechanical properties, such as its low elastic modulus, high flexibility, and high transparency [12]. Moreover, curing liquid PDMS for sensor fabrication improves its adhesion to the sensing layer, increasing sensor stability. Therefore, PDMS stretchable substrates have been studied extensively, and PDMS is a key material in stretchable strain sensor development [13,14,15,16].

Strain sensors are typically piezoresistive [17], capacitive [18], or piezoelectric [19]. In a piezoresistive strain sensor, the external strain is measured based on changes in the electric resistance of the sensing material. Therefore, both the substrate and the sensing material are key factors affecting the performance of a stretchable strain sensor. Many studies have demonstrated that nanomaterials, such as carbon nanotubes, carbon black, nanocarbon fibers, or graphene, can be used as sensing materials in strain sensors [9,20,21,22]. In particular, graphene has exceptional properties, such as high mechanical strength and high electrical conductivity, and thus has been identified as a promising material for strain sensors [23,24,25,26]. Different graphene preparation methods produce different graphene structures [27]. Some common graphene preparation methods are mechanical exfoliation, graphene oxide reduction, and chemical vapor deposition (CVD). However, these methods are typically both expensive and complicated. For example, in mechanical exfoliation, the first preparation method to be reported in the literature, graphene is produced by directly separating it from the surface of a graphite structure with an experimental instrument [28,29]. However, this process is difficult, time-consuming, and costly, and the quality of the produced graphene is inconsistent; thus, the method is unsuitable for large-scale graphene production. Reduced graphene oxide is prepared by using chemical or thermal reduction solution; the method can be used to continually produce homogenous graphene structures [30,31,32]. However, the large-scale preparation of reduced graphene oxide is limited by the fact that waste liquid from the preparation process is an environmental pollutant. The CVD process has recently been proposed for preparing high-quality graphene on an industrial scale [33,34,35]. However, the cost of the CVD equipment required for preparing large-area graphene films is high; thus, meeting the demand for products is challenging.

Laser-induced graphene (LIG) prepared with a laser scanning process has recently been proposed as a sensing material for strain sensors [36,37,38,39,40,41]. LIG, which has a three-dimensional (3D) porous network structure, is generated directly from polyimide (PI) film using a laser. The laser scanning process can be used to fabricate LIG patterns in a noncontact process via the laser-direct-write technique. Compared with other graphene preparation methods, laser induction can be used to fabricate and pattern graphene sensing materials in a simpler, lower-cost manner and is compatible with large-area graphene fabrication. Therefore, the working principles and electromechanical performance of LIG strain sensors have been widely studied in the literature. However, the effect of different laser-scanned LIG patterns on strain sensor performance has rarely been investigated.

**Figure 1:**
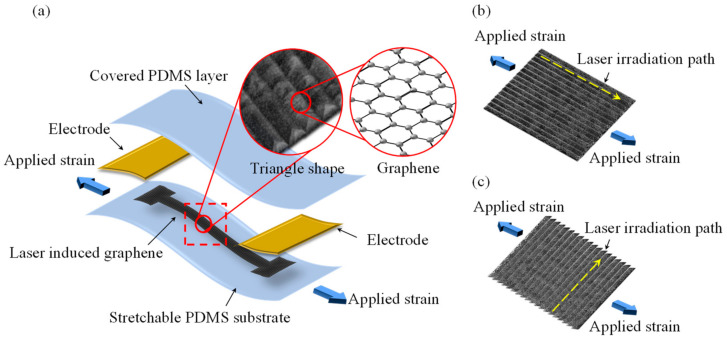
Schematic of the proposed (**a**) stretchable LIG strain sensor, (**b**) parallel LIG structure, and (**c**) vertical LIG structure.

In this work, a stretchable strain sensor based on a 3D porous LIG structure and PDMS was proposed and developed. A schematic of the proposed LIG stretchable strain sensor is displayed in Figure 1a. The LIG was used as the sensing material, and the elastic PDMS polymer was used as the substrate and covering layer. The sensing LIG structure was produced using CO_2_ infrared laser scanning and patterning on a PI film. Two laser irradiation paths were used to fabricate LIG structures with different stretching directions, namely parallel and vertical LIG structures, as presented in Figure 1b and Figure 1c, respectively. These different structures were characterized, and their performance was investigated. If the LIG strain sensor underwent an external displacement, the changes in electrical resistance were measured, and the applied stain was estimated.

## 2. Materials and Methods

### 2.1. Fabrication of the LIG Structure

**Figure 2:**
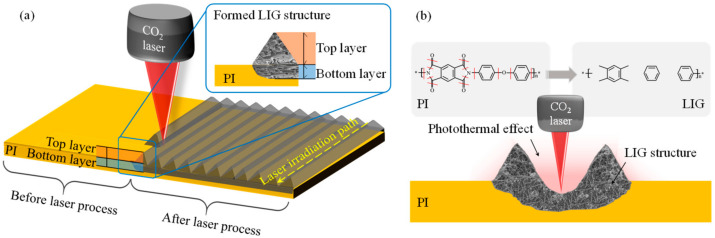
Schematic of (**a**) LIG fabrication by laser irradiation and (**b**) reaction principle of laser irradiation process.

A CO_2_ infrared laser was used to irradiate and scan a PI film to fabricate the LIG sensing structures, as shown in Figure 2a. To generate high-quality LIG structures, the laser irradiation was focused on the surface of the PI film to ensure that the irradiation was constant during the laser scanning process. When the laser irradiated the PI film, the photothermal effect was present on the film, as shown in Figure 2b [42,43,44,45]. Lattice vibration and lattice friction both increased the temperature at the irradiated region, and the resulting high temperature caused the chemical reactions that induced the formation of the LIG structure [36,46,47]. Chemical bonds in the PI film, such as C–O, C=O and C–N, broke at these temperatures [48], and the detached atoms recombined with O and N atoms in the ambient environment through oxidation reactions; the resulting gas products were released into the air. The LIG structure was formed directly by the carbonization of the residual carbon bonds. During the laser irradiation process, the highest temperature reached by the PI film was in the laser spot region. The temperature gradually decreased with distance from the laser spot. Triangular LIG structures were generated on both sides of the laser spot according to the variance in temperature distribution during the laser irradiation process. After the laser scanning process, the produced LIG structure was divided into a top and bottom layer. The top LIG layer was produced from the PI surface region. During its formation, the top LIG layer had sufficient space to expand and thus had a relatively loose structure. By contrast, the bottom LIG layer was formed deeper within the PI film and thus had a lower porosity [49].

### 2.2. Fabrication of the LIG Strain Sensor

**Figure 3:**
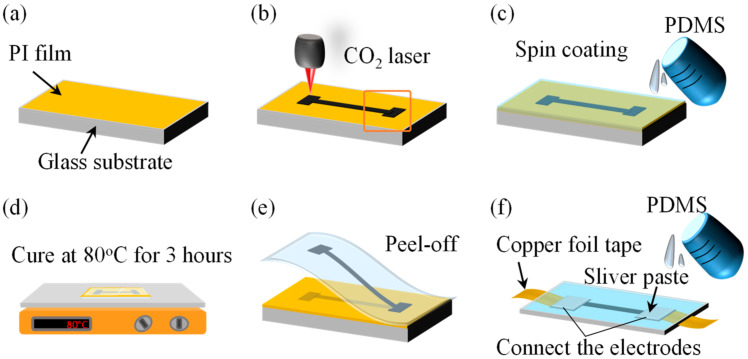
Fabrication process for the stretchable LIG strain sensor. (**a**) PI film was attached to the glass substrate, (**b**) a CO_2_ laser was used to scan on PI film, (**c**) PDMS was penetrate to LIG by spin coating, (**d**) PDMS was cured on hot plate, (**e**) PDMS film with an LIG layer was peeled from the PI film, (**f**) LIG strain sensor was prepared by connecting electrodes and PDMS covering process.

**Figure 4:**
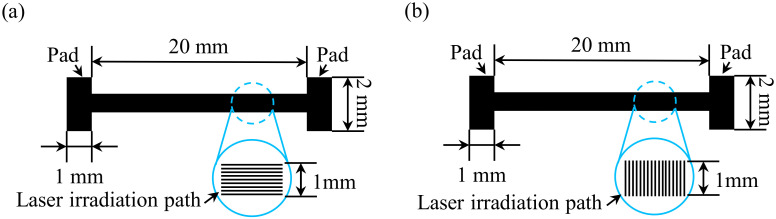
Laser scanning patterns. (**a**) Parallel and (**b**) vertical scanning.

The fabrication process for the stretchable strain sensor is presented in Figure 3. First, a 125 µm-thick polyimide (PI) film (UPI03-33, UNI FILM Co., Ltd., Taipei, Taiwan) was attached to a glass substrate (Figure 3a). A CO_2_ laser (LaserPro Venus II, GCC Innovation, New Taipei, Taiwan) was used to scan the PI film to produce the LIG structures (Figure 3b). The laser wavelength was 10.6 μm, and the laser spot diameter was 100 μm. The laser scanning parameters were as follows: scanning speed, 3 cm/s; laser power, 3.6 W; and scanning resolution, 500 dpi. The dimensions of the LIG strain sensor region were 20 mm in length and 1 mm in width (Figure 4). To produce two different LIG structures, laser scans with a parallel or vertical pattern were performed (Figure 4a and Figure 4b, respectively). The spacing between each line was set to 0.1 mm. After the laser scanning process, LIG had been generated on the PI film surface. PDMS was then spin-coated on the PI film with an LIG substrate surface at 300 rpm for 30 s to penetrate the LIG structure and form a thin film (Figure 3c). To prevent the formation of air bubbles in the LIG structure, a vacuum process was used to remove air bubbles from the PDMS. In addition, the vacuum process increased the ability of the PDMS to penetrate the LIG porous structure, stabilizing the LIG structure. The PDMS was then cured on a hot plate at 80 °C for 3 h (Figure 3d). After the curing process, the PDMS film with an LIG layer was peeled from the PI film (Figure 3e). If the PDMS did not penetrate the LIG structure, the LIG structure was destroyed during the peel-off process. Then, copper foil tape was stuck to the stretchable strain sensor to serve as the connecting electrodes. To decrease the contact resistance between the LIG and the copper foil tape, sliver paste was used as an interface material. Finally, a protective PDMS coating was applied to the strain sensor (Figure 3f).

### 2.3. Principles of the Strain Sensor

The piezoresistive strain sensor functions based on the change in the electric resistance of the sensing material in response to an external applied strain. For a sensing material, the resistance (*R*) can be expressed as follows:

(1) $$R=\frac{\rho l}{A}$$

Here, *ρ* is the resistivity, *l* is the length, and *A* is the cross-sectional area of the sensing material. If a piezoresistive material is deformed, such as due to an applied external strain, its resistance changes. For the LIG sensing material, the resistivity changes due to changes in the conductive path of the graphene resulting from deformation. The performance of the strain sensor can be evaluated in terms of the relationship between the relative resistance change and the applied strain; this relationship is indicated by the gauge factor (*GF*):

(2) $$GF=\frac{\Delta R/R_{0}}{\varepsilon },$$

Here, Δ*R* is the resistance change, *R*_0_ is the initial resistance, and *ε* is the applied strain. The gauge factor is a key index for the strain sensor. A larger gauge factor indicates that the strain sensor has higher sensitivity.

### 2.4. LIG Characterization and Strain Sensor Measurement Setup

Field emission scanning electron microscopy (FE-SEM, JSM-7800F, JEOL Ltd., Tokyo, Japan) was used to observe the surface morphology of the fabricated LIG structure and strain sensor. An X-ray energy dispersive spectrometer (EDS) was used to analyze the elemental content of the LIG structure to verify that the graphene was produced successfully from the PI. A Raman scattering spectroscopy system was used to analyze the material molecular composition. Because different carbon structures can be identified using the Raman peaks, Raman scattering spectroscopy was used to determine the structure of the fabricated LIG.

**Figure 5:**
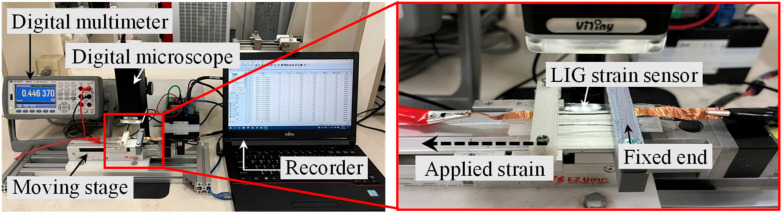
Measurement setup for the response of the stretchable LIG strain sensor.

To estimate the performance of the fabricated LIG strain sensor, a tensile testing measurement setup was developed (Figure 5). The fabricated LIG strain sensor was fixed to a fixed point and to a moving stage. A computer-controllable moving stage (EASM2NEX005, Oriental motor, Taichung, Taiwan) was used to displace the LIG strain sensor. A digital multimeter (GDM-8261A, Good Will Instrument, New Taipei, Taiwan) was connected to the strain sensor electrodes to record the resistance changes of the LIG strain sensor. A digital microscope (UM12, Microlinks, Kaohsiung, Taiwan) was used to observe the LIG structure during stretching.

## 3. Results and Discussion

### 3.1. Characteristics of LIG and LIG Sensor

**Figure 6:**
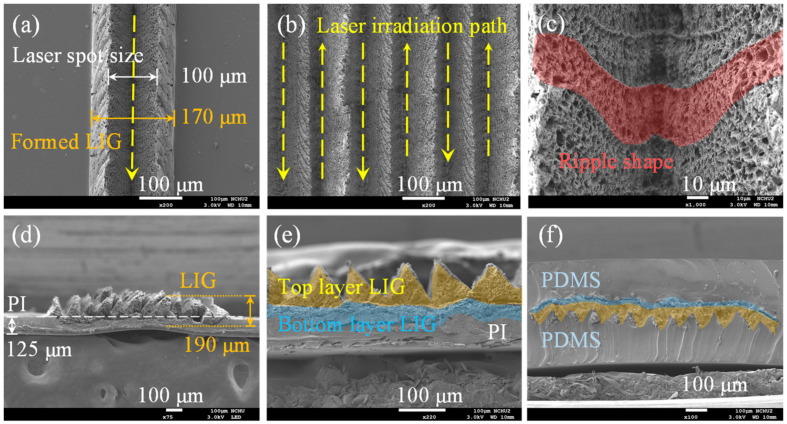
SEM images of (**a**–**e**) LIG structures formed by laser irradiation and (**f**) transfer of the LIG structure and packaging with PDMS.

SEM results on the morphology of the LIG structure revealed that the PI film was carbonized into a double-mountain LIG structure by the single-laser irradiation path (Figure 6a). Before laser irradiation, the PI film surface was smooth, without any visible impurities. The laser spot size was ≈100 µm, and the width of the formed LIG structure was ≈170 µm. Under laser irradiation, the PI material ablated and expanded from the laser spot to the surrounding region, causing gasification that resulted in the observed double-mountain, 3D, and porous LIG structure. The SEM images clearly reveal LIG structures with triangular mountain-like shapes (Figure 6b). The SEM image with higher magnification in Figure 6c reveals a rippled shape in the laser irradiation path, which indicates the overlap of each laser pulse. The thickness of the porous carbonized LIG structure was approximately 190 μm (Figure 6d). The formed LIG was divided into a top layer and a bottom layer (Figure 6e). The top-layer LIG structure had a triangular, mountain-like shape and higher porosity because the top layer had more space to expand during the formation process than the bottom layer did. The bottom LIG structure was a thin film with lower porosity due to the restricted space for expansion during laser scanning.

**Figure 7:**
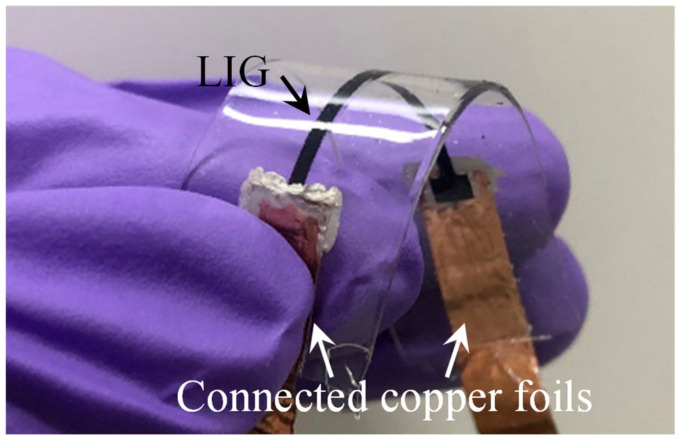
Fabricated LIG stain sensor.

After the LIG structure was transferred and packaged with PDMS, the original shape of the LIG structure was maintained (Figure 6f). The porous LIG structure was fully embedded in the PDMS layer; the other side was also coated with a PDMS layer to form a sandwich structure. The PDMS layers were used as a stretchable substrate to enhance the stability of the LIG sensing material. The total thickness of the produced LIG strain sensor was 500 μm. The fabricated LIG strain sensor had both good flexibility and good stretchability (Figure 7).

**Figure 8:**
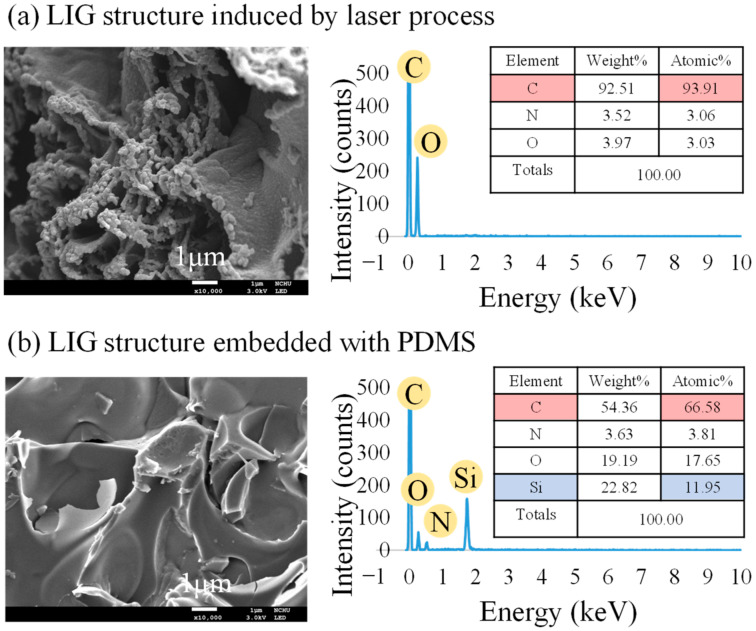
SEM images and EDS results for (**a**) the LIG structure and (**b**) the LIG structure embedded in PDMS.

The atomic content of the bare LIG structure and that embedded in PDMS were measured using EDS; the results are displayed in Figure 8a and Figure 8b, respectively. The carbon content ratio was 93.91% for the LIG structure, demonstrating that the LIG had both high carbonization and excellent quality [47]. High-magnification SEM images clearly revealed the 3D porous LIG structure. After the embedding in PDMS, the weight percentage of Si increased to 11.95% due to the composition of PDMS. The PDMS was fully embedded into the LIG structure due to the LIG structure’s porosity.

### 3.2. Raman Spectroscopy Results

Raman scattering spectroscopy was employed to evaluate the structural characteristics of the LIG. Three notable peaks for the LIG structures, namely D, G, and 2D, were observed at 1330, 1560, and 2680 cm^−1^, respectively [50]. The D peak indicates structural defects in the graphene structure, the G peak is the main characteristic peak reflecting the number of graphene layers, and the 2D peak can be used to verify the carbon material, indicating that graphene was formed and successfully embedded within the PI film. The intensity ratio of D/G indicates an inverse correlation with the degree of graphene crystallinity in the LIG; specifically, a higher D/G implies more numerous defects, lower crystallinity, and thus lower graphitization. Moreover, an intensity ratio of 2D/G > 2 indicates high-quality, single-layer graphene [51]; if 2D/G < 0.6, the produced graphene contains more than four layers. That is, a lower 2D/G ratio indicates a greater number of graphene layers. These Raman intensity ratios were used to compare the carbonization of the top and bottom layers of the LIG structure and to determine the quality of the LIG structure.

**Figure 9:**
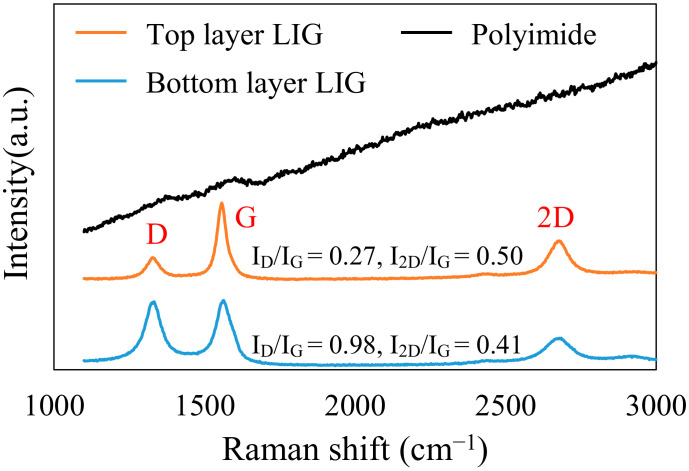
Raman scattering spectroscopy measurement results for the top- and bottom-layer LIG structures.

The Raman spectroscopy measurement results in Figure 9 reveal that both the top- and bottom-layer LIG structures are typical graphene structures. The D/G intensity ratios for the bottom and top layers were 0.98 and 0.27, respectively. The 2D/G intensity ratio for the top layer was higher, indicating that it had fewer defects and more layers than the bottom layer. Therefore, the top-layer LIG structure was more resistant to breakage due to the applied strain during the stretching process.

### 3.3. Performance of the LIG Strain Sensor

**Figure 10:**
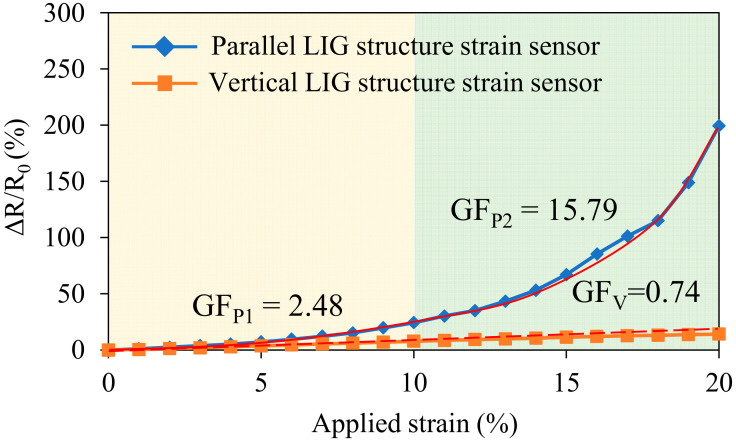
Relative resistance changes with different applied strain values for the parallel and vertical LIG strain sensors.

**Figure 11:**
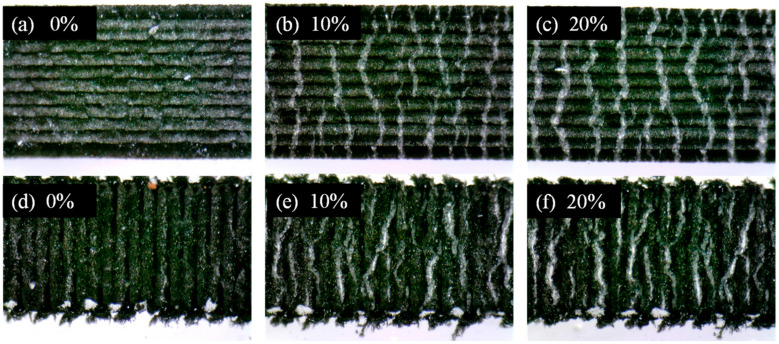
Digital microscope images of (**a**–**c**) parallel and (**d**–**f**) vertical LIG structures for applied strain values of 0%, 10%, and 20%, respectively.

A tensile test was performed to evaluate the performance of the parallel and vertical LIG strain sensors. The initial resistances of the two strain sensors before the application of strain were 0.92 and 6.04 kΩ for the parallel and vertical LIG strain sensors, respectively. The lower resistance of the parallel LIG strain sensor was attributed to the greater number of conductive paths in both the top- and bottom-layer LIG structures. For the vertical LIG strain sensor, the resistance was dependent on the minimum cross-sectional area. The minimum cross-sectional area of the vertical LIG strain sensor was the area between the mountain-like features. Therefore, the vertical LIG strain sensor had a higher resistance than the parallel sensor due to the lower number of conductive paths in the bottom-layer LIG structure of the vertical sensor. For the vertical LIG strain sensor, the conductive paths in the top layer had a smaller contribution to the electric resistance than those in the bottom layer. Various strain values were applied to each strain sensor, and the relative resistance changes were recorded. The measurement results are presented in Figure 10. The initial length of each strain sensor was 20 mm, and the maximum applied deformation was 4 mm, corresponding to an applied strain of 20%. When the LIG strain sensor underwent an applied tensile strain over 20%, the changes in the resistance of the LIG sensing layer were unstable. Sometimes the resistance was large and could not be measured. This was due to the excessive cracks that formed and the dramatically reduced conductive paths. The measurement results reveal that the gauge factor of the parallel LIG strain sensor varied with strain. The gauge factor of the parallel LIG strain sensor increased linearly between 0% and 10% applied strain (GF_P1_), reaching 2.48 at 10% applied strain. The gauge factor between 10% and 20% strain (GF_P2_) increased rapidly due to the rapid increase in the relative resistance, reaching a maximum of 15.79. The higher gauge factor observed for larger applied strain values was attributed to the decrease in the number of conductive paths, increasing the resistance (Figure 11a–c). By contrast, the resistance of the vertical LIG strain sensor increased slowly and linearly for all strain values during the tensile test. The gauge factor (GF_V_) was 0.74 throughout the applied strain range of 0% to 20%. This result reveals that the number of conductive paths in the bottom LIG structure of the vertical LIG strain sensor did not change substantially when strain was applied. The conductive paths of the vertical LIG strain sensor primarily relied on the bottom-layer LIG structures. Thus, the difference in the gauge factors of the parallel and vertical LIG strain sensors was attributed to the different LIG structure patterns.

A digital microscope was used to record images of the parallel and vertical LIG strain sensors during stretching (Figure 11a–c and Figure 11d–f, respectively). At higher strain values, cracks formed on the LIG sensing layer. The cracks were perpendicular to the applied strain loading, forming a striped pattern. The width of the formed cracks increased with the applied strain for strain values between 10% and 20%.

**Figure 12:**
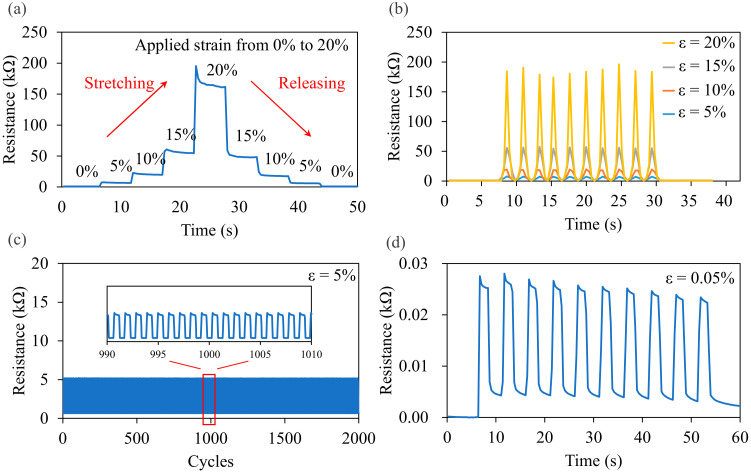
Performance of the parallel LIG strain sensor for (**a**) step response, (**b**) dynamic response, (**c**) durability testing, and (**d**) minimum sensing strain testing.

The performance of the parallel LIG strain sensor was investigated. The resistance of the parallel LIG strain sensor was measured at strain values of 5%, 10%, 15%, and 20% (Figure 12a). The stretching and releasing speeds were set to 1 mm/s, and the sensor was held at each strain for 5 s between each movement. The resistance changes for each applied strain were observed. The resistance returned to its initial value when the applied strain was released, indicating that the conductive paths in the parallel LIG structure were reformed after the release of the applied strain. The dynamic response measurements for four applied strain values are presented in Figure 12b. For applied strain values of 5%, 10%, and 15%, the changes in resistance were stable. However, a slight drift in resistance was observed every time a 20% strain was applied. This was attributed to the time required for the conductive paths to be rebuilt after being subjected to a 20% applied strain. This short-term unstable state was observable in the abrupt peak of the initial resistance after 20% strain was applied (Figure 12a). The durability testing result of the parallel LIG strain sensor for a 5% applied strain for 2000 stretch–release cycles is presented in Figure 12c. The resistance was stable after 2000 cycles. The parallel LIG strain sensor could sense a minimum applied strain of 0.05% (Figure 12d).

**Figure 13:**
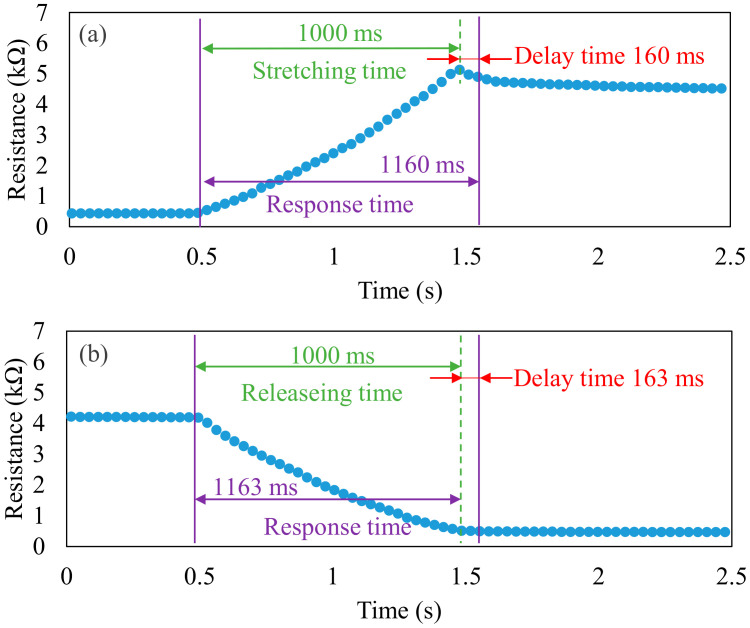
Response and delay times of the LIG strain sensor under an applied strain for (**a**) stretching and (**b**) releasing.

A strain sensor used in real-time monitoring must have a short response time. The response and recovery times of the parallel LIG strain sensor were related to the stretch and release times (Figure 13a and Figure 13b, respectively). In the tests, the deformation was set to 1 mm, corresponding to a 5% strain. The speed of the moving stage was 1 mm/s. Therefore, the 5% strain was applied and released with a 1000 ms duration. The resistance measurement results reveal that the response times for stretching and releasing were 1160 and 1163 ms, respectively. Therefore, compared with the durations of stretching and releasing, the recovery times were delayed by 160 and 163 ms, respectively. Thus, changes in resistance could be rapidly detected by the LIG strain sensor.

### 3.4. Demonstration of the LIG Strain Sensor

**Figure 14:**
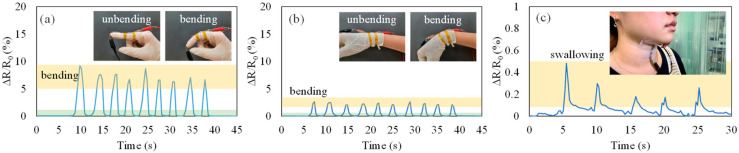
Detection of human motion using the developed LIG strain sensor for (**a**) finger-bending motion, (**b**) wrist-bending motion, and (**c**) throat-swallowing motion.

The developed LIG strain sensor was used to monitor human motion. The LIG strain sensor was fixed to a finger or wrist by tape to monitor bending by recording the relative resistance changes of the sensor. The measurement results for finger and wrist bending are presented in Figure 14a and Figure 14b, respectively. The relative changes in resistance clearly reflected the movements of the finger and wrist; the resistance increased as the finger or wrist bent and returned to its initial value when the finger or wrist returned to its initial state. In addition, the developed LIG strain sensor was fixed on the neck for monitoring the throat-swallowing motion, as shown in Figure 14c. Even though the throat-swallowing motion was very small in neck surface area, the LIG strain sensor was used to monitor it. Thus, the developed strain sensor could be used to monitor human motion.

## 4. Conclusions

In this study, a stretchable LIG strain sensor was proposed and developed. The LIG strain sensor comprised a LIG sensing material and a PDMS stretchable substrate. The LIG was prepared on a PI film using direct laser irradiation. The laser irradiation process could pattern the LIG structures in a large area, achieving simple and low-cost fabrication. Two different LIG pattern structures, parallel and vertical, were fabricated and used to produce LIG strain sensors. The laser-irradiated LIG structures could be divided into a top-layer LIG structure and a bottom-layer LIG structure. After they were embedded in PDMS, the conductive paths for these two layers differed. The parallel LIG strain sensor had a lower initial resistance and larger gauge factor than the vertical LIG structure, and the relative resistance changes of the parallel strain sensor were thus more easily observed than those of the vertical strain sensor for all strain ranges. The parallel LIG strain sensor had a high sensitivity, fast response time, excellent repeatability, and good durability. The maximum gauge factor of the parallel LIG strain sensor was 2.48 in the strain range of 0% to 10% and 15.79 in the strain range of 10% to 20%. The developed LIG strain sensor can thus be used to monitor human motions, such as finger bending, wrist bending, and throat swallowing, in real time.

## Data Availability

Not applicable.
